# True uterus didelphys in she-camel: a case report and review of literature

**DOI:** 10.3389/fvets.2024.1419234

**Published:** 2024-06-26

**Authors:** Mohamed A. A. Mahdy, Mohammed S. Nasr Eldeen

**Affiliations:** ^1^Department of Anatomy and Histology, Faculty of Veterinary Medicine, King Salman International University, Ras Sudr, Egypt; ^2^Department of Anatomy and Embryology, Faculty of Veterinary Medicine, South Valley University, Qena, Egypt

**Keywords:** congenital abnormalities, Müllerian duct anomalies, she-camel, uterus didelphys, case report

## Abstract

**Background:**

Uterus didelphys is a rare congenital anomaly of the female reproductive tract characterized by a divided uterine cervix and body. It occurs due to abnormal development of the paramesonephric (Müllerian) duct. Different forms of uterus didelphys have been reported in several animal species, including bovine, equine, ewe, goat, swine, and bitch. However, there is no previous report that has documented a completely divided female genital tract in she-camel. Moreover, there is a lack of literature regarding this anomaly in animals. Therefore, the present study reports, for the first time, a rare case of a completely divided female genital tract in a she-camel. In addition, the existing relevant literature on uterus didelphys in different animal species is reviewed.

**Case presentation:**

A female reproductive tract of she-camel, approximately 10 years old, with a history of previous successful pregnancy, was brought to the anatomy department following the slaughtering of the animal. Initial examination revealed a normal reproductive tract consisting of two ovaries, two fallopian tubes, a uterus, and a vagina. A closer examination revealed a completely divided vagina, with an external os opened into each part of the vagina, as well as a divided uterine body and cervix. Intrauterine infusion of saline through one external os confirmed complete separation of uterine body and cervix.

**Conclusion:**

To the authors’ knowledge, this is the first reported case of a completely divided female genital tract in a she-camel. This review summarizes the previous reports about uterus didelphys in farm animals.

## Introduction

Uterus didelphys is a rare congenital anatomical defect of the female reproductive tract of different animal species, including bovine, equine, sheep, goat, and pig (1–6). It happens due to the failure of fusion of the two paramesonephric (Müllerian) ducts, the primordium of the female reproductive tract. This congenital anomaly varies according to the degree of fusion failure of the two Müllerian ducts (7). A complete fusion failure of the two ducts results in uterus didelphys (true uterus didelphys), which is characterized by a completely divided genital tract including a double uterine body, two separated crevices, and a longitudinal vaginal septum (7–9). Partial fusion failure results in varying degrees of division affecting either the caudal part of the uterine body, cervix, or vagina, according to the area affected (7).

Abusineina (10) classified cervical abnormalities into four main types: uterus didelphys, complete double cervix, incomplete double cervix, and double external uterine orifices.

### Uterus didelphys

Uterus didelphys (completely divided female genital tract) is caused by the persistence of the median walls of the Müllerian ducts along their entire length, resulting in two cervices and two separate uterine bodies.

### Complete double cervix

It is caused by the persistence of the median walls of the Müllerian ducts along the whole length of the cervix, resulting in two cervices and one uterine body.

### Incomplete double cervix

It is caused by the persistence of the median walls of the Müllerian ducts at the posterior part of the cervix, resulting in one cervical canal cranially and two cervical canals caudally.

### Double external uterine orifices

It is caused by the persistence of the median walls of the Müllerian ducts at the external uterine orifice, resulting in one cervix with a band of tissue at the external os.

Different types of cervical abnormalities reported in different animal species are listed in Table 1. Clinical studies reported that congenital anomalies due to fusion failure of the Müllerian ducts are associated with reproductive difficulties (35). Ishiyama (36) reported that severe cases of incomplete fusion, such as double external os with blind diverticulum, complete double cervix with blind diverticulum, and uterus didelphys, are associated with infertility in cattle, while double cervix and external cervical os increase the incidence of dystocia in animals (33).

**Table 1:** Different reported types of cervical abnormalities in different animal species.

Anomaly	Species	Anatomical feature	Reference
Double cervix and double vagina	She-Camel	Double cervix and divided vagina	(11)
Uterus didelphys	Cow	Two cervices and two separate uterine bodies	(10, 12)
		Two cervices and two uterine bodies with divided cranial vagina	(2, 13)
Complete double cervix		Two cervices and one uterine body	(10, 14–17)
Incomplete double cervix		One cervix with a band of tissue divides the external os	(10)
Double external uterine orifices		Double cervix and divided vagina with normal uterus	(10)
Double cervix and double vagina		Double cervix and divided vagina with normal uterus	(18)
Uterus didelphys	Buffalo	Two cervices and two separate uterine bodies	(4, 19)
Double cervix		Double cervix, each cervix opens directly in the uterine horn with an absent uterine body and intercornual ligaments	(20, 21)
Uterus didelphys	Ewe	Two cervices and two uterine bodies with divided anterior vagina	(22, 23)
Complete double cervix		Two cervices and one uterine body	(24)
Double cervix and double uterine body		Two uterine bodies and two cervices with one cervix open into the common vestibule.	(25)
Uterus didelphys	Goat	Double cervix and divided uterine body	(26)
		Two cervices and two uterine bodies with divided anterior vagina	(3)
Double cervix and double vagina		Double vagina and cervices.	(26)
Double cervix	Mare	Two cervical canals	(27)
Double cervix and divided cranial vagina		Double cervix with divided anterior vagina and common uterine body.	(5)
Double cervix and double uterine body		Double cervix with divided uterine body	(28–30)
Double cervix	Sow	Completely divided cervix	(31)
Triple cervices and double uterine body		Three cervical canals open at the divided uterine body with two cervical canals open into the right side and one canal into the left side.	(6)
Double cervix and double vagina	Bitch	Separate uterine horns, double cervix, and two vaginal canals.	(32)
Double cervix and divided cranial vagina		Double cervix with divided cranial vagina and common uterine body.	(33)
Double vagina		Two vaginal canals and one vestibule	(34)

Although different forms of uterus didelphys have been reported in several animal species, only a case of double cervix and divided vagina has been reported in she-camel (11). There is no previous report that has documented a case of true uterus didelphys (completely divided female genital tract) in she-camel. Moreover, there is a lack of literature regarding this anomaly in animals. Therefore, the present study reports, for the first time, a rare case of a completely divided female genital tract in a she-camel. In addition to reviewing the existing relevant literature on uterus didelphys in different animal species, the current paper provides a brief summary of the anatomy, physiology, and development of the female reproductive tract in she-camels.

This condition is thought to be hereditary and associated with a recessive gene of unknown etiology. Cases of cervical duplication are detected incidentally at the time of breeding (33). The absence of Müllerian inhibiting substance (MIS) induces the differentiation of the Müllerian duct, forming the female reproductive tract (37). The cranial part that runs parallel to the mesonephric ducts and the transverse part that crosses the mesonephric duct develop, forming the epithelium of the fallopian tubes. The medial walls of the caudal fused part degenerate, forming a single canal (38) which develops into the epithelium of the uterus and cranial vagina (39). The species-specific morphological characteristics of the uterus (either simplex, bicornuate, or duplex) depend on the degree of fusion of Müllerian ducts being either complete, partial, or incomplete (38). The differentiation of Müllerian ducts is regulated by the members of Hoxa genes, specifically Hoxa9, Hoxa10, Hoxa11, and Hoxa13 (8, 37). The morphological diversity in the uterus among different animal species is due to different expression levels of Hoxa13 and Hoxd13gene (38). Other genes involved in the development of the Müllerian ducts include Emx2, Pax2, Lim1, and members of Wnt family 4 (Wnt4, Wnt5a, and Wnt7a) (8, 37, 40, 41).

## Case presentation

**Figure 1:**
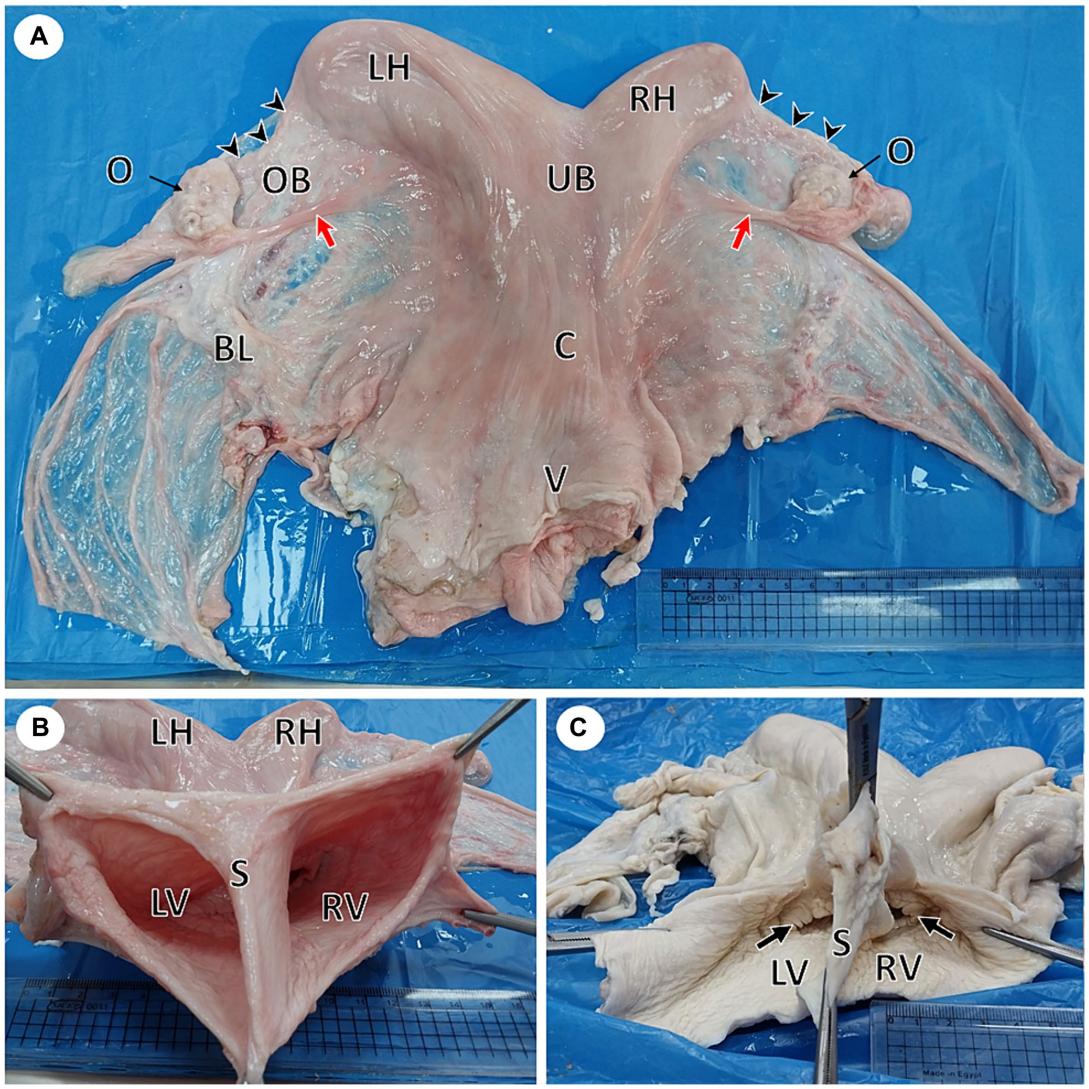
Gross photographs of female reproductive system of a she-camel show: **(A)** Apparently normal reproductive system consisted of right and left ovaries (O), each ovary was located inside an ovarian bursa (OB), the ovary attached to the uterine horn by the round ligament (red arrow), fallopian tubes (arrowheads), right (RH) and left (LH) horns, uterine body (UB), cervix (C), and vagina (V). Note the broad ligament of the uterus (BL). **(B)** Per vaginal view shows a completely divided vagina into the left (LV) and right (RV) vagina by a median septum (S). **(C)** The dorsal wall of the vagina was opened, showing each vagina had its external os (arrow).

A female reproductive tract of she-camel was brought to the anatomy department, Faculty of Veterinary Medicine, King Salman International University, Egypt, following slaughter at an abattoir in Giza governorate. Consultation with the owner revealed that the animal’s weight was approximately 270 kg and aged approximately 10 years old, with a history of previous successful pregnancy. Initial gross examination revealed a normal reproductive tract consisting of two ovaries and two flexuous fallopian tubes; each tube opened into a uterine horn. The two ovaries were normal and functional, as evidenced by the presence of some growing follicles on the left ovary and corpus luteum on the right one. The two horns were attached to a uterine body, followed by a cervix and vagina with no oblivious marks externally. The vulva and the caudal part of the vagina had been cut away. Closer examination of the reproductive tract revealed the presence of a completely divided vagina. Each division of the vagina had an external os opened into it (Figure 1). Morphometric measurements of the different parts of the female reproductive tract in this case were taken using a caliper. The data are listed in Table 2.

**Table 2:** Morphometric measurements of the female reproductive tract of the reported case.

Dimensions	Measurements (mm)
Right ovary: –Length –Width Left ovary:–Length. –Width	26 22 36 22
Right Fallopian tube length Left Fallopian tube length.	144 161
Right uterine horn: –Length –Width at the upper third –Width at the middle third –Width at the lower third Left uterine horn:–Length – Width at the upper third –Width at the middle third – Width at the lower third	68 26 38 46 112 33 46 58
Width of the uterine body	78
Right vagina width	62
Left vagina width	75

**Figure 2:**
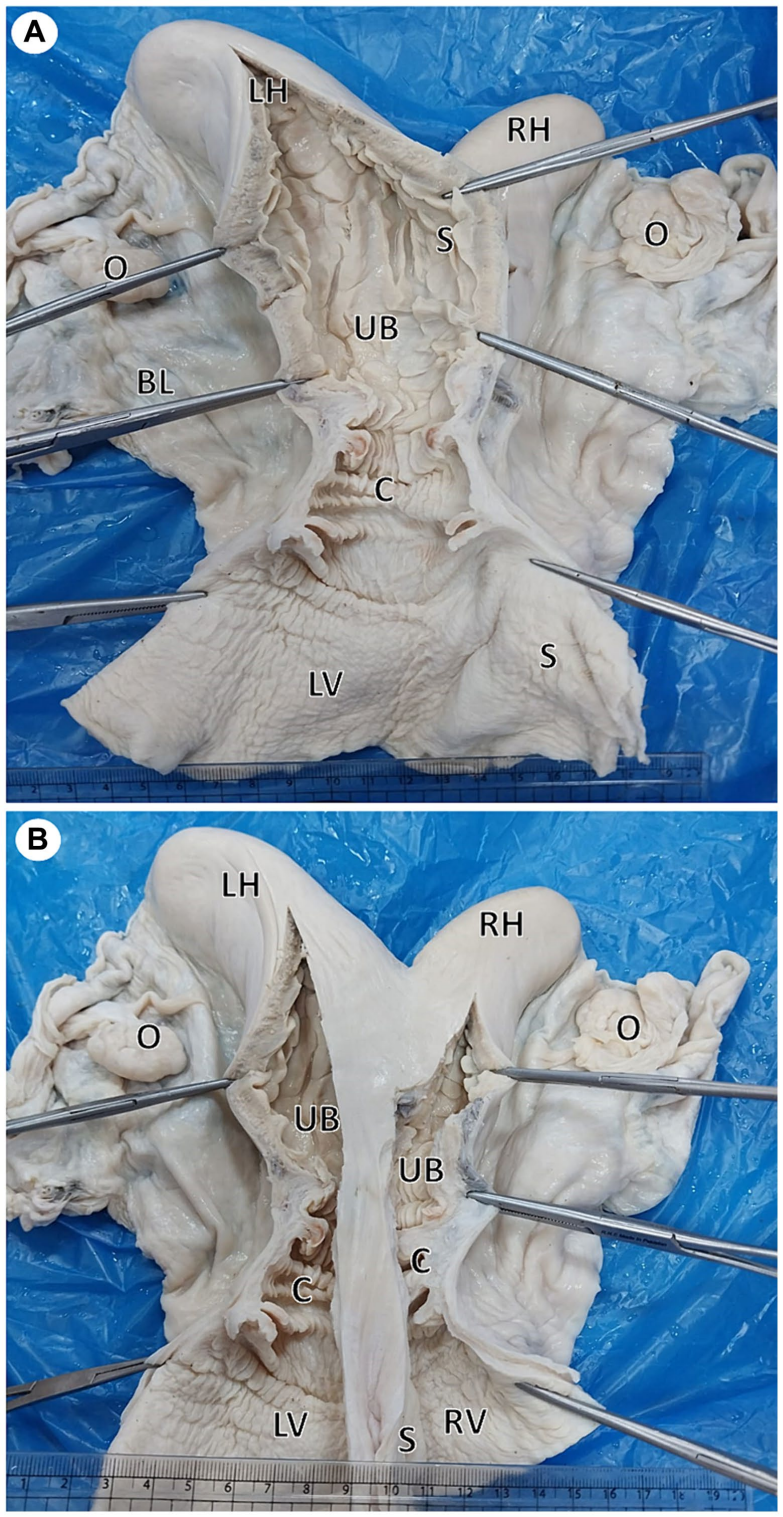
Gross photographs showing a dorsal view of uterus didelphys: **(A)** An incision was performed in the left horn (LH), body (UB), cervix (C), and vagina (LV). Note the complete septum extending from the fundus to the vagina. **(B)** Incised right (RH) and left (LH) horns show each horn connected to a separate uterine body that had its own internal and external os.

Intrauterine infusion of saline solution through the left external os revealed complete separation of uterine body and cervix. The uterus was then preserved in 10% neutrally buffered formalin. Later, a longitudinal incision was performed through each uterine horn, passing through the uterine body, cervix, and vagina. A complete longitudinal septum extending from the fundus to the vagina was observed. Each horn was connected to a separate uterine body that had its own internal and external os (Figure 2).

## Discussion

The female reproductive tract develops from the paramesonephric (Müllerian) ducts. The Müllerian duct appears as a longitudinal invagination of the genital ridge lateral to the mesonephric (Wolffian) ducts. This invagination deepens and then separates from the peritoneal lining, forming a solid cord, which canalizes later (37, 39). The Müllerian ducts run lateral and parallel to the Wolffian ducts, with their cranial ends opening into the coelomic cavity with a funnel-like structure. The ducts pass caudomedially and ventrally, crossing the mesonephric ducts to fuse, forming the uterovaginal duct, with its caudal end projecting into the urogenital sinus, forming the Müllerian tubercle (8, 39).

Uterus didelphys is a rare congenital anomaly of the female reproductive tract that has been reported in different animal species (3, 4, 19, 20, 24, 25). Abusineina (10) classified cervical abnormalities into four main types: uterus didelphys, complete double cervix, incomplete double cervix, and double external uterine orifices. In the present case, the uterus was completely divided by a longitudinal septum, resulting in two uterine bodies, two separated cervices with their own internal and external os, and a completely divided vagina, indicating a case of uterus didelphys.

The condition has been attributed to the failure of fusion of the two paramesonephric (Müllerian) ducts. This congenital anomaly varies according to the degree of fusion failure of the two ducts (7). A complete fusion failure of the two ducts results in a double uterine body, the uterus didelphys. In this case, each uterine horn opens into a separate uterine body that leads to two separate crevices with a longitudinal vaginal septum (7–9).

The present case had functional ovaries, as indicated by the presence of growing follicles on the left ovary and a corpus luteum on the right one, which indicated normal cyclic activity of the ovary. Clinical studies have reported that congenital anomalies due to the fusion failure of the Müllerian ducts are associated with reproductive difficulties (35). Ishiyama, Nakamura (36) reported that severe cases of incomplete fusion, such as double external os with a blind diverticulum, complete double cervix with blind diverticulum, and uterus didelphys, are associated with infertility in cattle, while the double cervix and external cervical os increase the incidence of dystocia in animals (9, 33). Uterine anomalies restrict uterine space, which either alters or decreases the efficiency of the functional placenta, causing fetal growth deficiency (42). Moreover, artificial insemination is considered a challenge as the semen might be deposited in the cervix on the side opposite to the ovary from which ovulation has occurred. However, a cow with uterus didelphys had a normal birth after semen was introduced into each cervical canal (14). The present case had a history of previous successful pregnancies following natural mating and normal parturition. In accordance with the present data, reports claim that animals with uterus didelphys undergo a normal conception rate following natural mating (9, 20). Moreover, pregnancy has been reported in a cow (12) and an ewe (24) with uterus didelphys. Chethan and Singh (20) claimed that the condition is hereditary and associated with recessive genes of unknown etiology. Therefore, such animals should be excluded from breeding after diagnosis.

Uterus didelphys can be diagnosed during the pre-breeding examination, through a variety of methods, including physical examination using vaginoscopy, endoscopy, transrectal ultrasonography, and intrauterine injection of saline through one cervix to visualize the two separate uterine bodies with the median septum in between (28).

Molecular (DNA) marker-assisted selection and cytogenetic analyses linked to genes of interest with significant impacts on reproduction, such as WNT and HOXA genes, can be reinforced for the early selection of breeding females. This would aid in the development of cytogenetic profiles and molecular markers for diagnosing reproductive diseases with a genetic or physiological origin. This, in turn, would allow for the detection of animals with reproductive disorders and enable culling at an early stage (43).

In conclusion, the present study reported, for the first time, a rare case of uterus didelphys in a she-camel. The uterine body, cervix, and vagina were divided completely by a longitudinal septum extended from the fundus to the vagina. This rare case may have an educational role, either for the students studying the congenital abnormalities of the reproductive tract or for the practitioner/technician in the field, who should be alert for the pre-breeding diagnosis of conditions that may hinder female fertility. Moreover, the current study provides an overview of uterus didelphys in farm animals.

## Data availability statement

The original contributions presented in the study are included in the article/supplementary material, further inquiries can be directed to the corresponding author.

## Ethics statement

Ethical approval was not required for the study involving animals in accordance with the local legislation and institutional requirements because the study was done on slaughterhouse materials, so no need for ethical approval. Written informed consent was obtained from the owners of the animals for the publication of this case report.

## Author contributions

MM: Writing – review & editing, Writing – original draft, Visualization, Validation, Supervision, Software, Project administration, Methodology, Investigation, Formal analysis, Data curation, Conceptualization. MN: Writing – review & editing, Writing – original draft, Visualization, Methodology, Investigation, Formal analysis.
